# Supplementary material for: Genetic basis of falling risk susceptibility in the UK Biobank Study
Source: Commun Biol. 2020 Sep 30;3:543. doi: 10.1038/s42003-020-01256-x (PMC7527955; doi:10.1038/s42003-020-01256-x)
Supplement: Supplementary file 1 — Supplementary Information [file 42003_2020_1256_MOESM1_ESM.docx]

**Supplementary Figures**


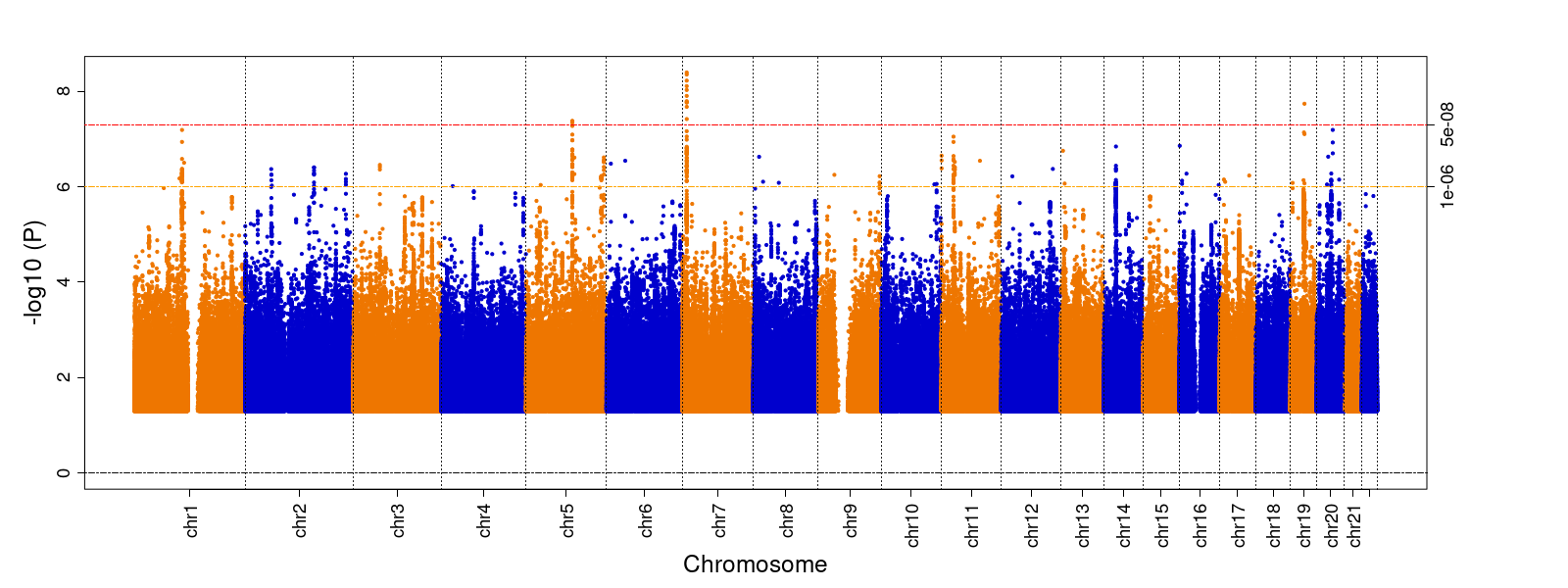


**Supplementary Figure 1.** Manhattan Plot of Association Statistics (−log_10_(P)) for falling risk for the combined meta-analysis. Each dot represents a SNP and the x axis indicates its chromosomal position (built 37 NCBI). Dashed horizontal red line marks the GWS threshold (P ≤5 × 10^−8^).


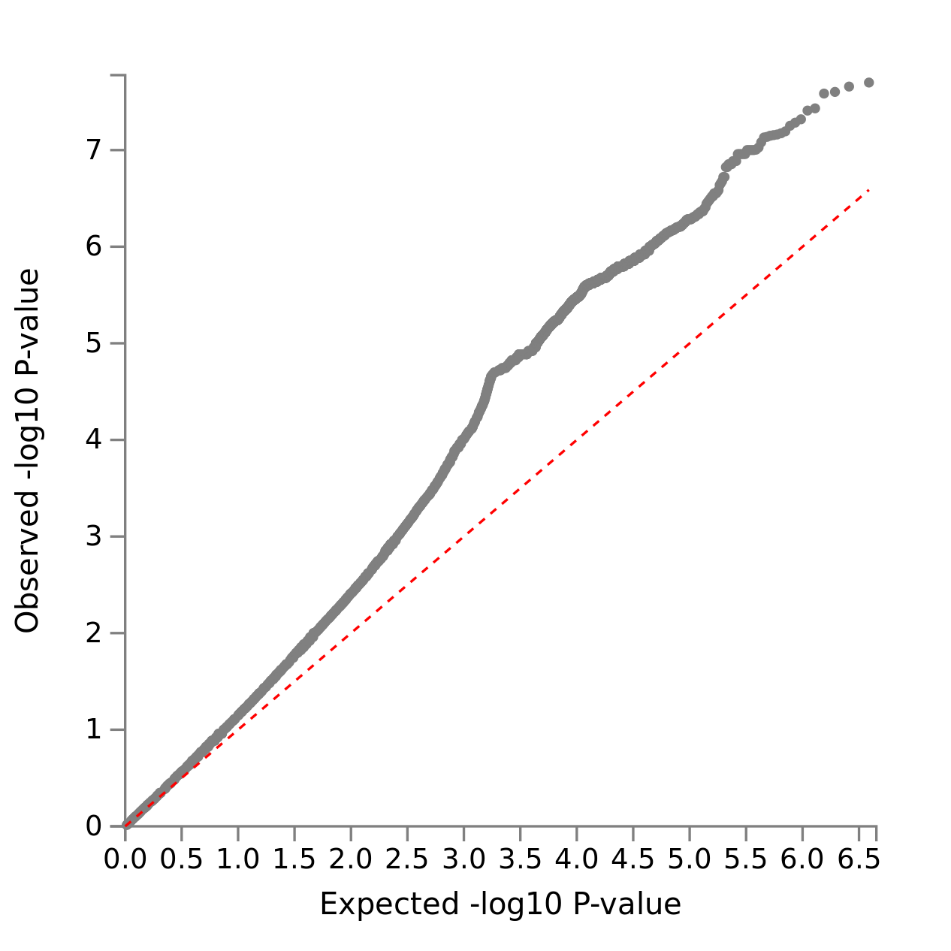


**Supplementary Figure 2**. Quantile-quantile (Q-Q) plot of observed versus expected P values of the GWAS results. The straight line in the Q-Q plot indicates the distribution of SNPs under the null hypothesis

**
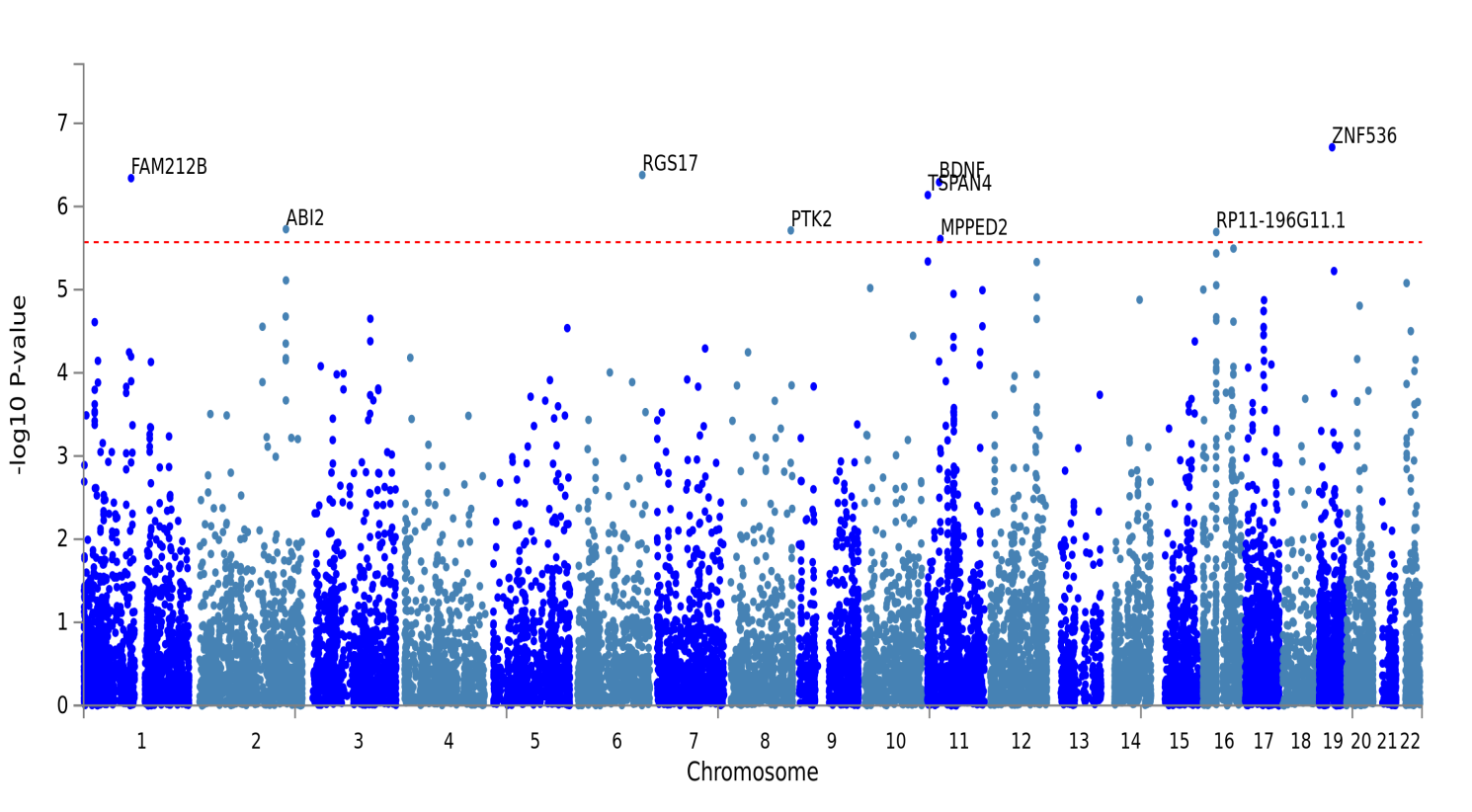
**

**Supplementary Figure 3.** Manhattan plot of the gene-based test as computed by MAGMA. Each dot represents one gene and the x axis indicates its chromosomal position (built 37 NCBI). The dashed red horizontal line marks the gene-number-adjusted threshold (p=0.05/18,185 tested genes).


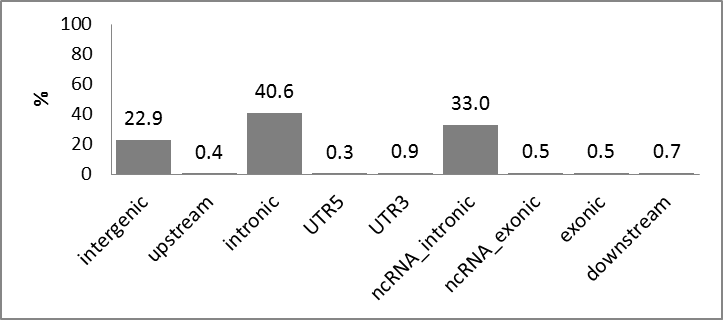


**a**


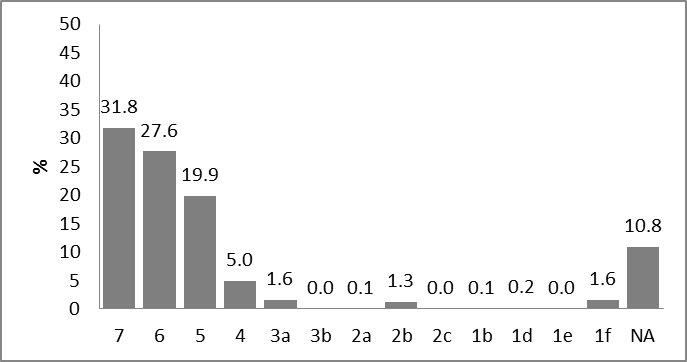


**b**


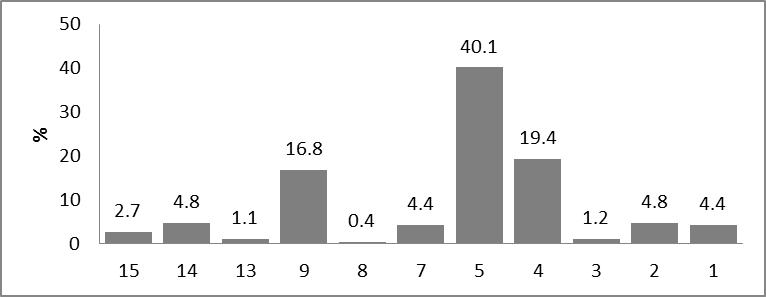


**c**

**Supplementary figure 4.** Functional annotation for all SNPs with r^2^ ≥ 0.6 with the top SNPs (P<1x10^-6^). a) Percentage of SNPs according to their functional category; b) Percentage of SNPs according to their RegulomeDB score (x-axis). Lower score indicates a more likely regulatory role; c) Percentage of SNPs according to their minimum chromatin state across 127 tissues. Lower score (x-axis) indicates a more likely regulatory role. NA – not available in RegulomeDB.

**a**


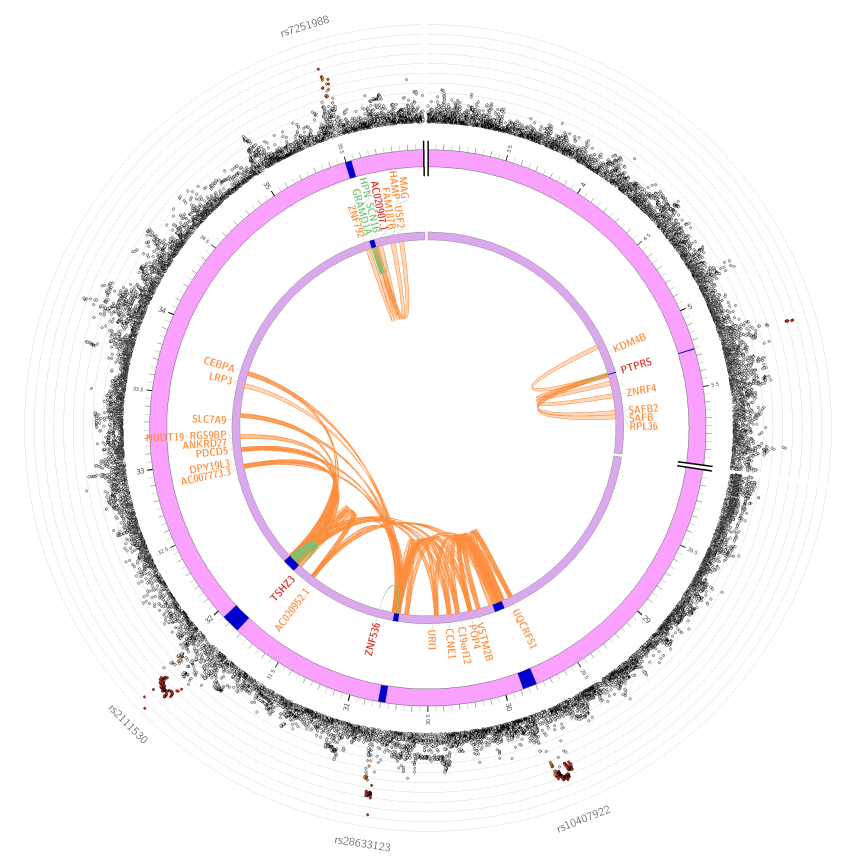


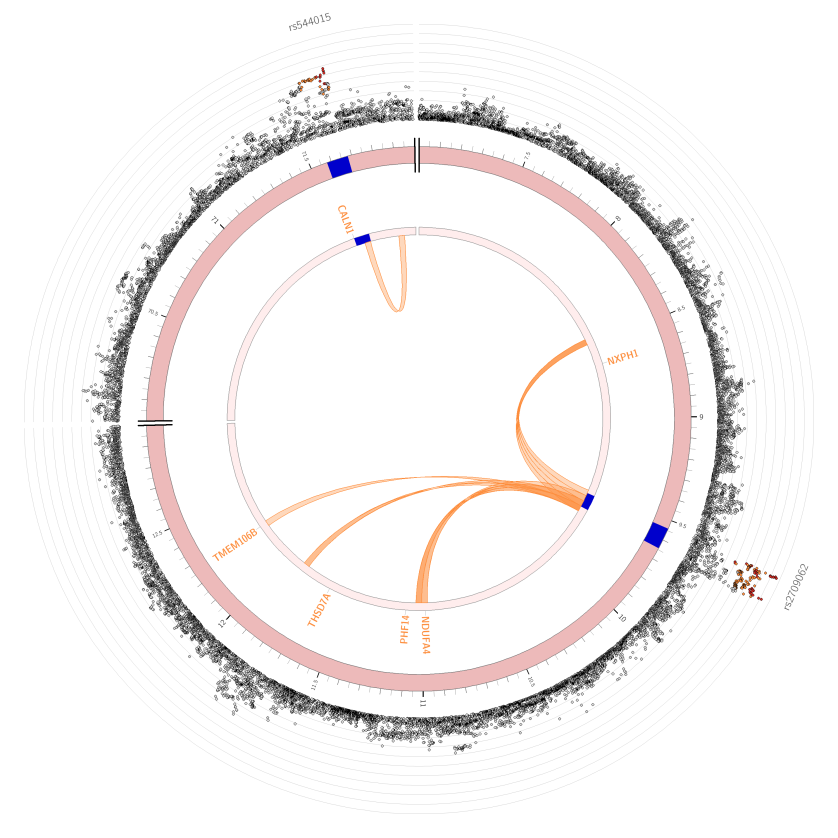


**b**


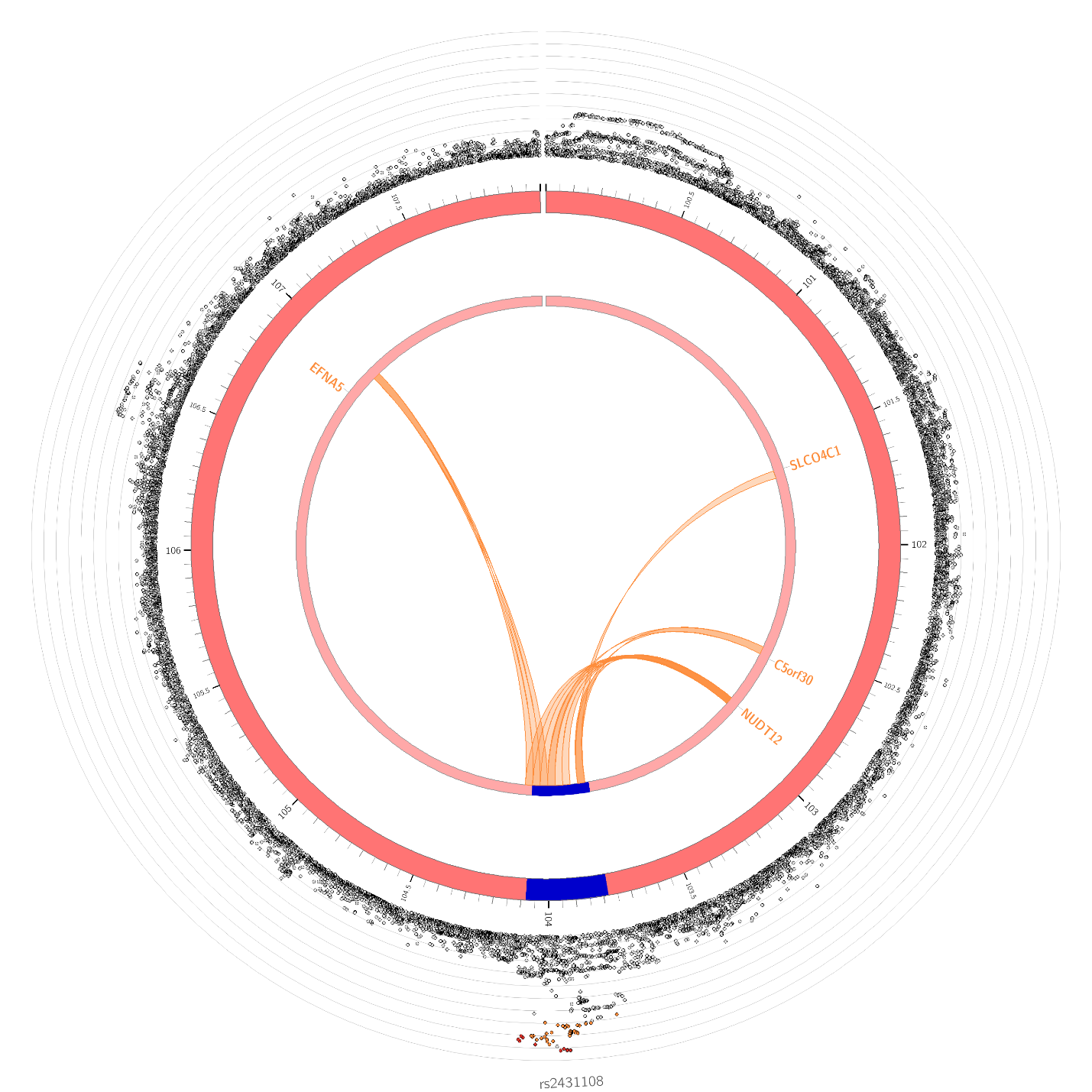


**c)**

**Supplementary Figure 5.** Circos plots demonstrating the results of eQTL and chromatin interaction mapping for loci on chromosome **a)** 7, **b)** 19 and **b)** 5. Genes mapped by Hi-C or eQTLs are colored orange and green, respectively. Genes that were mapped by both eQTL associations and chromatin interaction data are highlighted in red. The highlighted SNPs (blue box) represent the GWS SNPs (P ≤5 × 10^−8^).

**Supplementary Tables**

**Supplementary Table 1.** Previously reported SNP- and Gene-associations from the GWAS catalog

| **SNP** | **Know SNP-associations** | **GENE** | **Know Gene-associations** |
| --- | --- | --- | --- |
| rs2431108 | Insomnia, Anxiety, Nap during the day, Leg fat percentage, Neurotism, Well-being, Loneliness, Depression, Mornigness | *RP11-6N13.1* | - |
| rs2709062 | - | *PER4* | - |
| rs2111530 | Systolic blood pressure | *TSHZ3* | Lung function, Chronotype measurement, BMD |
| rs76259395 | - | *NTNG1* | Blood proteins, LDL-cholesterol, BMI |
| rs6658723 | BMI, Fat Mass, Waist Circumference, Waist-Hip ratio | *FAM212B* | BMI* |
| rs67174662 | BMI, Fat Mass, Waist Circumference | *EIF3FP3* | Neuroticism, Mood swings |
| rs974135 | Height | *BCL11A* | Blood traits, Education, Cognition |
| rs7616516 | - | *FHIT* | BMI, Physical activity, Smoking, Depression |
| rs2471020 | - | *DRD1* | Eye refractive error |
| rs72857666 | - | *TRERF1* | Blood traits |
| rs12666565 | - | *NXPH1* | chronotype measurement |
| rs11030084 | BMI, Fat Mass, Fat-free Mass, Hip and Waist Circumference, Menarche, Smoking, Risk-taking behaviour | *BDNF-AS* | BMI*, Hip* and Waist circumference, Menarche*, chronotype measurement, smoking*, alcohol consumption |
| rs494221 | Height, Hip circumference, Fat-Free Mass, Basal metabolic rate | *MPPED2* | Estimated glomerular filtration rate, height, Weight circumference, chronotype measurement |
| rs28672671 | - | *TSPAN4* |  |
| rs12884871 | - | *RPL10L* | BMI |
| rs28633123 | - | *ZNF536* | Height, BMI, education |
| rs6063547 | - | *CTNNBL1* | - |

*the lead SNPs for both traits are in LD

**Supplementary Table 2.** Mendelian Randomization analyses of several potential risk factors for falls

|  | **IVW** |  | **Weighted median** |  | **MR-Egger** |  | **Egger Intercept** | **Number of SNPs** |
| --- | --- | --- | --- | --- | --- | --- | --- | --- |
|  | *OR (95%CI)* | *P* | *OR (95%CI)* | *P* | *OR (95%CI)* | *P* | *P* | *N* |
| Relative handgrip | 0.41 (0.23 to 0.41) | <0.0001 | 0.44 (0.23 to 0.85) | <0.0001 | 0.24 (0.02 to 2.09) | 0.197 | 0.63 | 103 |
| Body mass index | 1.13 (1.06 to 1.20) | <0.0001 | 1.16 (1.08 to 1.24) | <0.0001 | 1.25 (1.08 to 1.44) | 0.002 | 0.12 | 77 |
| Alcohol consumption | 1.01 (0.99 to 1.04) | 0.38 | 1.00 (0.97 to 1.02) | 0.82 | 1.01 (0.94 to 1.08) | 0.84 | 0.91 | 97 |
| Alcohol dependence | 1.04 (1.01 to 1.08) | 0.029 | - |  | - |  | - | 1 |
| Antihypertensive drugs |  |  |  |  |  |  |  |  |
| ACE inhibitors | 1.00 (0.97-1.03) | 0.80 | - |  | - |  | - | 1 |
| Beta-blockers | 0.99 (0.97-1.01) | 0.47 | 0.99 (0.98-1.01) | 0.37 | 1.03 (0.96-1.10) | 0.45 | 0.30 | 6 |
| Calcium channel blockers | 1.00 (0.99-1.01) | 0.43 | 0.99 (0.97-1.01) | 0.46 | 0.99 (0.97-1.00) | 0.16 | 0.23 | 24 |

Footnote: The weighted median and the Egger regression MR analyses require at least 3 SNPs for implementation. The OR for relative hand grip (m^2^), BMI (kg/m^2^) and alcohol consumption (log transformed) are per 1unit increase in the exposure.
