# Supplementary material for: Genetic basis of falling risk susceptibility in the UK Biobank Study
Source: Commun Biol. 2020 Sep 30;3:543. doi: 10.1038/s42003-020-01256-x (PMC7527955; doi:10.1038/s42003-020-01256-x)
Supplement: Supplementary file 2 — Description of Additional Supplementary Files [file 42003_2020_1256_MOESM2_ESM.pdf]

Description of Additional Supplementary Files

File name: Supplementary Data 1
Description: Association of falls PRS adjusted for age and sex across 17 different p-value thresholds (from 5x10-8 to 1) within two different populations (B-PROOF study falls calendar and falls questionnaire, and Rotterdam Study falls questionnaire)

File name: Supplementary Data 2
Description: Estimates of the genetic correlation between falls and different traits and Medications

File name: Supplementary Data 3
Description: Heritability partitioning enrichment estimates across tissue groups using LDSEG

File name: Supplementary Data 4
Description: Tissue enrichment analysis estimates using MAGMA
