# Supplementary material for: Genetic basis of falling risk susceptibility in the UK Biobank Study
Source: Commun Biol. 2020 Sep 30;3:543. doi: 10.1038/s42003-020-01256-x (PMC7527955; doi:10.1038/s42003-020-01256-x)
Supplement: Supplementary file 4 — Reporting Summary [file 42003_2020_1256_MOESM4_ESM.pdf]

nature research

Corresponding author(s): Fernando Rivadeneira

Last updated by author(s): Jul 6, 2020

Reporting Summary

Nature Research wishes to improve the reproducibility of the work that we publish. This form provides structure for consistency and transparency in reporting. For further information on Nature Research policies, see Authors & Referees and the Editorial Policy Checklist .

Statistics

For all statistical analyses, confirm that the following items are present in the figure legend, table legend, main text, or Methods section.

n/a	Confirmed	
☐	☒	The exact sample size (*n*) for each experimental group/condition, given as a discrete number and unit of measurement
☐	☒	A statement on whether measurements were taken from distinct samples or whether the same sample was measured repeatedly
☐	☒	The statistical test(s) used AND whether they are one- or two-sided<br>*Only common tests should be described solely by name; describe more complex techniques in the Methods section.*
☐	☒	A description of all covariates tested
☐	☒	A description of any assumptions or corrections, such as tests of normality and adjustment for multiple comparisons
☐	☒	A full description of the statistical parameters including central tendency (e.g. means) or other basic estimates (e.g. regression coefficient) AND variation (e.g. standard deviation) or associated estimates of uncertainty (e.g. confidence intervals)
☐	☒	For null hypothesis testing, the test statistic (e.g. *F*, *t*, *r*) with confidence intervals, effect sizes, degrees of freedom and *P* value noted<br>*Give P values as exact values whenever suitable.*
☒	☐	For Bayesian analysis, information on the choice of priors and Markov chain Monte Carlo settings
☒	☐	For hierarchical and complex designs, identification of the appropriate level for tests and full reporting of outcomes
☐	☒	Estimates of effect sizes (e.g. Cohen's *d*, Pearson's *r*), indicating how they were calculated

Our web collection on statistics for biologists contains articles on many of the points above.

Software and code

Policy information about availability of computer code

Data collection	UK-Biobank
Data analysis	Genetic association analyses were performed using BOLT-LMM (https://data.broadinstitute.org/alkesgroup/BOLT-LMM/)<br>Functional annotations were done using FUMA (https://fuma.ctglab.nl/)<br>Polygenic risk scores were calculated using PRSice (http://www.prsice.info/)<br>The Mendelian Randomization analyses were done using the R-package MendelianRandomization (https://cran.r-project.org/web/packages/MendelianRandomization/index.html)<br>SNP heritability and genetic correlations were estimated using LD score regression (https://github.com/bulik/ldsc) and LD hub (http://ldsc.broadinstitute.org/).<br>Stratified heritability and functional enrichment and tissue specificity analyses were done using LD score regression to specifically expressed genes (LDSC-SEG) (http://ldsc.broadinstitute.org/).

For manuscripts utilizing custom algorithms or software that are central to the research but not yet described in published literature, software must be made available to editors/reviewers. We strongly encourage code deposition in a community repository (e.g. GitHub). See the Nature Research guidelines for submitting code & software for further information.

Data

Policy information about availability of data

All manuscripts must include a data availability statement. This statement should provide the following information, where applicable:

- Accession codes, unique identifiers, or web links for publicly available datasets
- A list of figures that have associated raw data
- A description of any restrictions on data availability

Genotype and phenotype data from UKBB have already been processed and are available at https://www.ukbiobank.ac.uk/. Data access have been granted to11 application number 24268. Data from the Rotterdam Study and BPROOF are available on request from the corresponding author.

Field-specific reporting

Please select the one below that is the best fit for your research. If you are not sure, read the appropriate sections before making your selection.

- [x] Life sciences
- [ ] Behavioural & social sciences
- [ ] Ecological, evolutionary & environmental sciences

For a reference copy of the document with all sections, see nature.com/documents/nr-reporting-summary-flat.pdf

Life sciences study design

All studies must disclose on these points even when the disclosure is negative.

Sample size	We performed the largest possible GWAS that can be drawn within the UK Biobank which is powerful enough to detect novel signals.
Data exclusions	Samples not passing the GWAS quality control thresholds (data quality, ancestry and relatedness) were excluded. In addition, samples with missing phenotypes (fall information) were not included in the analysis. Full description is provided in the methods section.
Replication	Replication was performed using two independent cohorts with fall information. Full description is provided in the methods section.
Randomization	Not applicable (not an experimental design)
Blinding	Not applicable (not an experimental design)

Reporting for specific materials, systems and methods

We require information from authors about some types of materials, experimental systems and methods used in many studies. Here, indicate whether each material, system or method listed is relevant to your study. If you are not sure if a list item applies to your research, read the appropriate section before selecting a response.

Materials & experimental systems

n/a	Involved in the study	
x		Antibodies
x		Eukaryotic cell lines
x		Palaeontology
x		Animals and other organisms
	x	Human research participants
x		Clinical data

Methods

n/a	Involved in the study	
x		ChIP-seq
x		Flow cytometry
x		MRI-based neuroimaging

Human research participants

Policy information about studies involving human research participants

Population characteristics	Our analyses were performed using data from the UK Biobank study which is a large prospective cohort study of approximately a half-million adult (ages 40-69) participants living in the United Kingdom (UK), Detailed description is provided in the methods section.
Recruitment	The UK Biobank participants were recruited from 2 centers across the UK in 2006-2010.
Ethics oversight	Ethical approval was granted by the Northwest Multi-centre Research Ethics Committee.

Note that full information on the approval of the study protocol must also be provided in the manuscript.
